# Supplementary material for: Proteomics Analysis of Extracellular Matrix Remodeling During Zebrafish Heart Regeneration
Source: Mol Cell Proteomics. 2019 Jun 20;18(9):1745–55. doi: 10.1074/mcp.RA118.001193 (PMC6731076; doi:10.1074/mcp.RA118.001193)
Supplement: Supplementary Material [file 141786_2_supp_341119_psdtf2.pdf]

Supplementary Information

Proteomics analysis of extracellular matrix remodeling during zebrafish heart regeneration

Anna Garcia-Puig[1,2], Jose Luis Mosquera[3], Senda Jiménez-Delgado[1], Cristina García-Pastor[1], Ignasi Jorba[4,5,6], Daniel Navajas[4,5,6], Francesc Canals[7], and Angel Raya[1,2,8,]*

[1] Center of Regenerative Medicine in Barcelona (CMRB), 3rd Floor Hospital Duran i Reynals, Avinguda de la Gran Via 199-203, 08908 Hospitalet de Llobregat (Barcelona), Spain.

[2] Center for Networked Biomedical Research on Bioengineering, Biomaterials and Nanomedicine (CIBER-BBN).

[3] Bioinformatics Unit, Institut d'Investigació Biomèdica de Bellvitge IDIBELL), 3rd Floor Hospital Duran i Reynals, Avinguda de la Gran Via 199-203, 08908 Hospitalet de Llobregat (Barcelona), Spain.

[4] Institute for Bioengineering of Catalonia (IBEC), Barcelona Science Park, Baldiri Reixac 15-21, 08028 Barcelona, Spain.

[5] Unit of Biophysics and Bioengineering, Department of Physiological Sciences I, School of Medicine, University of Barcelona.

[6] Center for Networked Biomedical Research on Respiratory Diseases (CIBERES).

[7] Proteomics group, Vall d'Hebron Institut of Oncology (VHIO), Cellex center, Natzaret 115-117, 08035 Barcelona, Spain.

[8] Institució Catalana de Recerca i Estudis Avançats (ICREA).

*Address for correspondence: Angel Raya, Phone: +34 933 160 320; Fax: +34 933 160 332; Email: araya@cmrb.eu

Running title: Regenerating heart ECM proteome

Keywords: extracellular matrix; heart regeneration; proteomic analysis; atomic force microscopy.

Supplementary Information contains:

7 Supplementary Tables as individual sheets in attached Excel file:

Supplementary Table 1. Proteins identified during the decellularization process. Native zebrafish hearts, after SDS treatment (half), and at the end of decellularization (fully), were analyzed by LC-MS. Log2 raw intensities and VSN normalized intensities are given for each replicate of each sample group.

Supplementary Table 2. Peptides identified in the decellularized samples. Table of all the entries identified in our samples. The table contains the protein name, accession number, molecular weight and the spectral counts values obtained per each replicate.

Supplementary Table 3. List of proteins represented with an overall of >5 spectral counts. The table contains for each protein the gene name, the accession number, the molecular weight (MW), the ANOVA value (F), the p-value, and the adjusted p-value (FDR), the values obtained per each replicate and the average. The ECM proteins are identified with an asterisk.

Supplementary Table 4. List of proteins represented with an overall of >5 spectral counts, normalized by VSN method. The table contains for each protein the gene name, the accession number, the molecular weight (MW), the ANOVA value (F), the p-value, and the adjusted p-value (FDR), the values obtained per each replicate and the average. The ECM proteins are identified with an asterisk.

Supplementary Table 5. List of proteins differentially expressed during heart regeneration. The table contains for each protein the gene name, the accession number, the molecular weight (MW), the ANOVA value (F), the p-value, and the adjusted p-value (FDR), the values obtained per each replicate and the average. The ECM proteins are identified with an asterisk. p-value obtained from an ANOVA test of all the samples.

Supplementary Table 6. List of the peptide identification in LC-MS analysis.

Supplementary Table 7. List of protein identifications.

1 Supplementary Figure

Supplementary Figure 1. Biological Process GO enrichment analysis on the differentially expressed proteins during cardiac regeneration. Comparison of the enriched Biological Process GO terms on our differentially expressed proteins (**A**) and the 209 differential protein groups of Ma D. et al. (**B**).

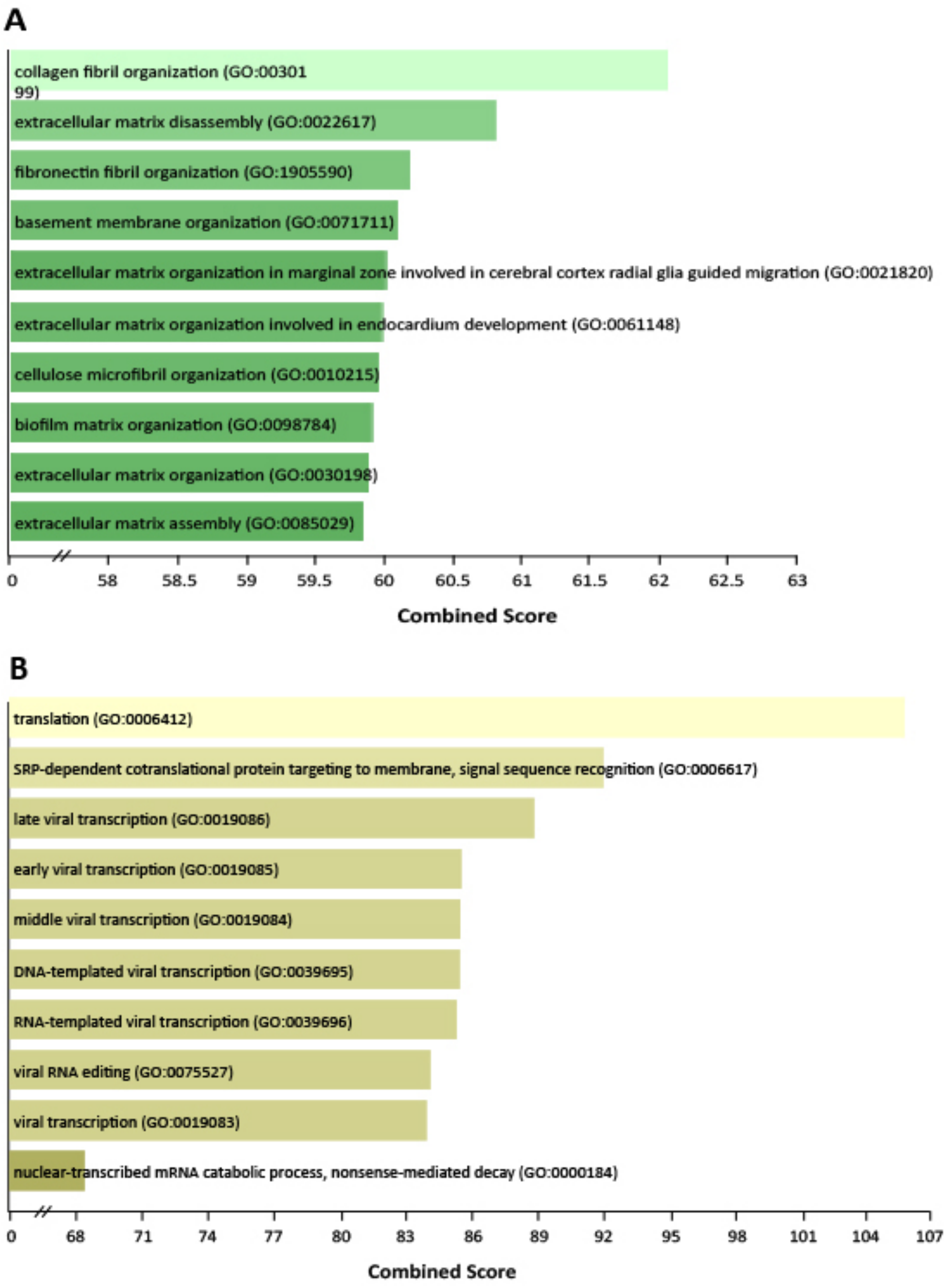

Supplementary Fig. 1. Biological Process GO enrichment analysis on the differentially-expressed proteins during cardiac regeneration Comparison of the enriched Biological Process GO terms on our differentially expressed proteins (A) and the 209 differential protein groups of Ma et al.[53] (B).
